# Endotoxin induces conversion of endothelial cells into activated fibroblasts

**DOI:** 10.1186/cc12977

**Published:** 2013-11-05

**Authors:** César Echeverría, Ignacio Montorfano, Daniela Sarmiento, Alvaro Becerra, Claudio Cabello-Verrugio, Felipe Simon

**Affiliations:** 1 Facultad de Ciencias Biológicas, Universidad Andres Bello, Santiago, Chile; 2 Millennium Institute on Immunology and Immunotherapy, Santiago, Chile

## Background

Endothelial dysfunction is a key step in endotoxemia-derived sepsis syndrome pathogenesis. It is well accepted that the bacterial endotoxin lipopolysaccharide (LPS) induces endothelial cell (EC) dysfunction through immune system overactivation [1-3]. However, LPS can also affect ECs in the absence of participation by immune cells [4-6]. Although interactions between LPS and ECs evoke endothelial death, a significant portion of ECs are resistant to LPS challenge [6-8]. However, the mechanism that confers endothelial resistance to LPS is not known. Considering that LPS-resistant ECs exhibit a fibroblast-like morphology, suggesting that these ECs enter in a fibrotic program in response to LPS, our aim was to investigate whether LPS induces endothelial fibrosis and explore the underlying mechanism.

## Materials and methods

We used two different models: primary ECs, and intact blood vessels (IBV). Both preparations were freshly obtained from umbilical cord veins from normal pregnancies, after patients' informed consent. The investigation conforms with the principles outlined in the Declaration of Helsinki. The Commission of Bioethics and Biosafety of Universidad Andres Bello approved all experimental protocols. Once the preparation was established they were cultured with or without LPS as a model of endotoxemia. ECs were exposed to 20 mg/ml LPS for 72 hours, while IBV was challenged to 20 mg/ml LPS on the inside for 48 hours.

## Results

ECs exposed to LPS showed a fibroblast-like morphologic change. In addition, LPS-treated ECs showed an upregulation of both fibroblast-specific protein expression such as fibroblast specific protein-1 and α-smooth muscle actin, and extracellular matrix proteins secretion including fibronectin and collagen type III. In concordance, ECs exposed to endotoxin showed a severe downregulation of endothelial markers such as vascular endothelial cadherin and the platelet endothelial cell adhesion molecule-1 (CD31). Similar results were obtained in the endothelial monolayer from IBV perfused with LPS in which abundant fibrosis was observed. Furthermore, we demonstrate that LPS-induced EC fibrosis is dependent on the endotoxin receptor toll-like receptor-4. In addition, the participation of NAD(P)H oxidase activity and ROS generation was demonstrated using specific blockers. Finally, we demonstrated by means of small interfering technology and a pharmacological inhibitor that LPS-induced EC fibrosis is dependent on the activin like kinase-5 kinase activity, suggesting that tumor growth factor beta is involved in this fibrotic process.

## Conclusions

We conclude that LPS is able and sufficient to promote endothelial fibrosis. It is noteworthy that LPS-induced endothelial fibrosis perpetuates endothelial dysfunction as a maladaptive process rather than a survival mechanism for protection against LPS. These findings are useful in improving current treatment against endotoxemia-derived sepsis syndrome and other inflammatory diseases.
